# Combined Diagnostic Modalities Improve Detection of Detrusor External Sphincter Dyssynergia

**DOI:** 10.5402/2011/323421

**Published:** 2011-11-22

**Authors:** Sara Spettel, Carmin Kalorin, Elise De

**Affiliations:** ^1^Albany Medical College, 43 New Scotland Avenue, Albany, NY 12208, USA; ^2^Continence Center, Urological Institute of Northeast New York, NY 12209, USA; ^3^Division of Urology, Albany Medical College, 23 Hackett Boulevard, Albany, NY 12208, USA

## Abstract

*Introduction*. The diagnosis of detrusor-external sphincter dyssynergia (DESD) is a clinically relevant finding during urodynamic testing. However, there is no consensus regarding diagnostic specifics of electromyography (EMG) or voiding cystourethrography (VCUG). We evaluated the concordance of the two modalities most commonly used in clinical practice for the diagnosis of DESD. *Methods*. Patients were prospectively evaluated by a single urodynamicist at an academic center and retrospectively re-evaluated by an independent urodynamicist for agreement. DESD was determined by increased patch EMG activity or a dilated bladder neck/proximal urethra on VCUG during detrusor contraction. Minimal acceptable criterion for agreement was set at 70%. *Results*. Forty-six patients were diagnosed with DESD with both modalities available. Of these 46 patients, 25 were diagnosed by both tests, 11 by VCUG alone and 10 by patch EMG alone. Binomial testing demonstrated the proportion of agreement was 54% (95% CI 39% to 68%). *Conclusion*. We found significant disagreement between the two modalities, similar to previously reported findings using needle EMG, and we expand the applicability of our data to the majority of clinicians who use patch EMG electrodes. This further supports the idea that the combined use of EMG and VCUG for diagnosis can identify more cases of DESD than either modality alone.

## 1. Introduction

Urodynamic testing has greatly improved our diagnostic capabilities for patients with complex neurogenic bladder dysfunction. However, despite years of refinements in technical standards and recommendations, urodynamics require experience-based individual interpretation and lack established sensitivity and specificity data.

Detrusor external sphincter dyssynergia (DESD) is defined as: “a detrusor contraction concurrent with an involuntary contraction of the urethral and/or periurethral striated muscle” [1] in a patient with a known neurologic condition. Diagnosis is made by a combination of a clinical history of a known or potential upper tract lesion, increase in visual or audible electromyography (wire or patch), fluoroscopic visualization of a dilated bladder neck and proximal urethra to the external urethral sphincter (EUS), or a urethral pressure profile with interruption of flow (when present) with increased detrusor pressure during interruption.

Most urodynamic centers in the United States employ patch electrodes rather than needles for electromyography. The International Continence Society (ICS) has published statements in order to standardize reporting of many components of urodynamic results and technique [2, 3]. Certain parameters for diagnosis of DESD have been noted, such as an electromyography (EMG) recording minimum of 20 kHz [3], and the suggestion that quantitative measurement may be supplemented by imaging (videourodynamics). However, standard methodology and recommendations such as the type of needle and needle versus surface patch electrodes for the recording of external sphincter activity has not been published. The importance of each diagnostic test or parameter is left to the individual physician and published studies vary widely in EMG technique and reporting.

Some of the discordance between variables is intuitive; for example, a closed bladder neck on voiding cystourethrography (VCUG) prohibits visualization of the external urethral sphincter. In the case of EMG, electrode placement is operator dependent and the patch introduces additional factors due to interference from the perianal musculature.

To evaluate the concordance of the two modalities most commonly used in clinical practice, we prospectively examined the question of diagnostic discordance employing patch EMG compared to VCUG for the diagnosis of DESD.

## 2. Material and Methods

Institutional review board approval was obtained for the investigation. Patients were prospectively evaluated by a single urodynamicist (ED) and entered into an institutional database over a 24-month period. This database represented all patients offered urodynamics in an academic referral-based continence center. The urodynamic readings were then retrospectively re-evaluated by an independent urodynamicist (CK) for agreement with the original diagnosis of DESD.

The presence of DESD was determined by

**Figure 1:**
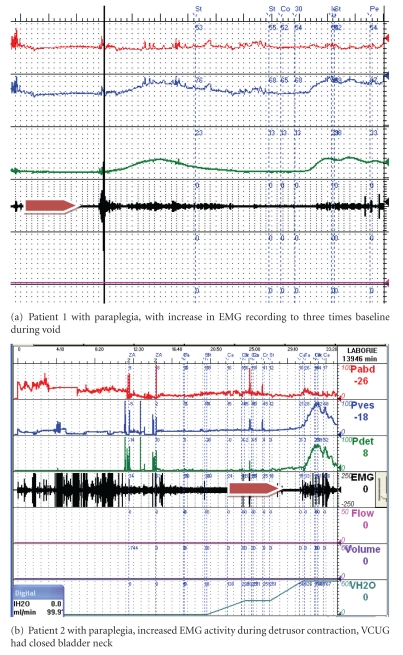
Electromyography.

**Figure 2:**
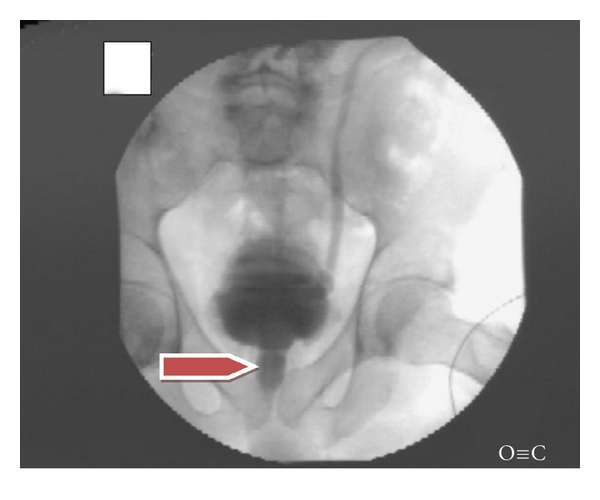
Voiding cystourethrogram: Patient 3 with spinal cord injury showing a dilated proximal urethra to external urethral sphincter (arrow), also left ureteral reflux.

1. increased patch EMG activity and/or (Figure 1),
2. a dilated bladder neck and proximal urethra on multichannel videourodynamics during detrusor contraction (Figure 2).

In the absence of valsalva, pelvic floor dystonia (dysfunctional voiding), or attempt to inhibit voiding, for example guarding during uninhibited contraction (UIC). Isolated mild EMG elevation in the absence of flow interruption, elevated detrusor pressure, or neurological disease was interpreted as dysfunctional voiding. Patients with history of sphincterotomy or urethral stent or whose urodynamics lacked the relevant data points (e.g., omission of fluoroscopy in severe contrast allergy) were excluded from analysis. The urethral pressure profile was examined for increase in detrusor pressure and drop in flow when present. Additional data points analyzed include age, sex, supine position, permissive voiding or UIC, postvoid residual, other source of obstruction, and neurologic diagnosis.

Urodynamics were meticulously performed according to the International Continence Society guidelines and recorded using multichannel technique on the Laborie Triton PRO 835 Urodynamics System, LAB850 Urodynamics Software (Laborie Medical Technologies Inc., Willistion, Vt, USA). The primary urodynamicist was present for all studies. Briefly, two patch EMG electrodes (Cleartrode, ConMed Corp., Utica, NY, USA [2]) were placed at the 2 and 10 o'clock position around the anus and a third ground patch electrode was placed over the adductor tendon on the medial aspect of the patient's left knee [1]. The electrodes were then covered with tape to prevent them from becoming wet or dislodged. A dual lumen 8-french urodynamic catheter (C.R. Bard, Inc., Covington, Ga, USA) was primed with contrast and, using aseptic technique, was introduced per urethra [3]. Transducers were leveled at the pubic symphysis. A rectal subtraction balloon catheter was then placed per rectum with 2.5 cc in the 10 cc balloon and taped in position (CAT510 Silicone Rectal Catheters, Laborie Medical Technologies Inc., Willistion, VT, USA). After confirming brisk and yoked response of the catheters to cough, the system was zeroed to atmospheric pressure. The volume in the rectal balloon was adjusted to achieve a detrusor pressure (Pdet) of between 0 and 4 cmH20. Filling cystometry was initiated using contrast at a rate of 30 cubic centimeters per minute. Sensation, when present, was recorded. Leak was recorded when present. Detrusor contraction was observed fluoroscopically and simultaneous patch EMG recording was obtained. Detrusor-external sphincter dyssynergia was diagnosed as detailed above. 

Minimal acceptable criterion for agreement between the EMG and VCUG was set at 70% concordance. Binomial test was employed to establish the presence or absence of agreement. For continuous variables the comparisons between groups was calculated by the nonparametric Kruskal-Wallis test for data not normally distributed. 

## 3. Results

Of the 376 patients prospectively entered into the database 52 were given a diagnosis of DESD. After retrospective review of readings, 6 were eliminated for having only one modality available (e.g., noncontrast exam) or other source of obstruction (e.g., urethral stricture), leaving 46 patients with DESD and no other source of obstruction. Of these 46 patients, 25 were diagnosed by both tests, 11 by VCUG alone and 10 by patch EMG alone (Table 1).

The agreement between the two diagnostic modalities was compared, assuming that the patients identified in the database adequately represent patients diagnosed with DESD by patch EMG and VCUG in a referral clinical practice. Setting a minimal acceptable criterion of 70% agreement for two diagnostic tests, we found that patch EMG and VCUG do not agree for a positive diagnosis of DESD. Binomial testing demonstrated significant disagreement (*P* = 0.02) in observed proportions. The proportion of agreement was 54% (95% CI 39% to 68%), which is significantly less than our criteria for agreement of 70% (Table 2).

Patients with DESD had a statistically higher PVR (214 ± 33) compared to patients without DESD (56 ± 22, *P* = 0.001) and higher mean detrusor pressure (47.3 ± 6.3) versus (33.4 ± 3.5, *P* = 0.045) during contraction. 

Of these 46 patients with DESD, exactly 50% were male compared to 29% male in the entire urodynamics database. Of the two tests, male patients were more likely to be diagnosed by EMG alone, while female patients were more likely to be diagnosed by VCUG alone. (Table 1) The average age of patients with DESD was (49 ± 16), not statistically different from the general database (56 ± 15).

In the recordings, a pressure flow variation was clearly seen in 14 tracings, unobtainable (due to supine position or lack of flow) in 13, obscured in 6 and not present in 4 patients assigned a diagnosis of DESD. 

The neurologic diagnoses are presented in Table 3 with some patients carrying more than 1 diagnosis. The most common diagnoses were multiple sclerosis (13) and spinal cord injury or disease (11 total, 7 with paraplegia and 4 with quadriplegia).

## 4. Discussion

Our study was designed to provide information about the diagnostic congruence of the two most commonly used tests performed in the United States for diagnosis of DESD during urodynamic testing. We are not promoting surface electrodes as a recommended modality for diagnosis but intended it would be most useful to investigate what our patients are seeing in practice. Our findings demonstrate a 54% agreement and 46% disagreement between patch EMG and VCUG for diagnosing DESD. The visualization of the external sphincter by VCUG is precluded by bladder neck dyssynergy or outlet obstruction from the prostate, which would explain the higher incidence in male patients. Likewise artifact from the urinary stream tracking down the perineum can preclude a clear EMG reading in female patients. 

The discordance found in this study between patch EMG and VCUG in a voiding dysfunction practice is similar to our previous results with wire needle EMG in a rehabilitation hospital practice, which demonstrated a 60% agreement and 40% disagreement for DESD by VCUG [4]. While previous studies have shown improved EMG measurements using needles [5], patch EMG is more widely used due to patient tolerance, ease of electrode placement and greater freedom of movement. Our previous paper discusses the wide variation in type of EMG used throughout the urodynamic literature, including the limitations of in use of the external anal sphincter as a proxy [4, 6–10]. It is not the aim of the current study to compare or assess the validity of two approaches but rather to point out that there is significant discordance between VCUG and the most commonly used EMG technique for diagnosis of DESD.

Technical factors with EUS EMG placement are of course suggested in the cases lacking and EMG diagnosis. It is well known that patch EMG suffers from many limitations even when identified and addressed. Most urodynamicists pay attention to multiple additional factors during interpretation including suspicion for the diagnosis (presence of an upper motor neuron lesion interrupting the spinobulbar pathways), obstruction of flow (if the patient leaks or voids), elevated detrusor pressure (diagnosis of DESD implies high voiding or storage pressures and potential for damage to the upper urinary tracts) and increased auditory feedback for those who perform oscilloscopy. The technologies for diagnosis are only tools to inform the observer.

We argue that interpretation is aided by the presence of the urodynamicist during the procedure. For example, if there is an uninhibited contraction and EMG/EUS tone increases, the increase could be due to voluntary guarding. Increased EUS activity during detrusor contraction in a neurologically intact woman who is nervous about the test is interpreted differently than increased activity in a patient with quadriplegia and no bladder sensation in this example. The examiner can instruct the sensate patient to relax the pelvic floor and allow a void during the next urge on repeat cystometrogram. The permissive void will allow for better assessment of EUS coordination.

In this and the 2005 article regarding wire needle EMG, the significant amount of disagreement between modalities suggests that the routine combination of EMG and VCUG will identify more cases of DESD than either modality alone. Index of suspicion is essential to prevent false diagnoses. We include our data on voiding pressures and flow interruption to support the diagnosis of DESD. These types of observations also inform the clinical impression. Given the nature of urodynamic testing, it is unlikely a gold standard could be agreed upon for the diagnosis of DESD. Better understanding of the interplay and relative importance of the techniques should be pursued as guidelines to the benefits and limitations of each modality contributing to the diagnosis could improve objective assessment of DESD.

## 5. Conclusions

In this prospective study employing consistent technique and using patch EMG electrodes, we found only 54% (95% CI 39% to 68%) agreement between patch EMG and VCUG in the diagnosis of DESD. This reinforces our prior similar findings using needle EMG and expands the applicability of our data to the majority of clinicians who use patch EMG electrodes. This further supports the idea that the combined use of EMG and VCUG or other modalities for diagnosis can identify more cases of DESD than either modality alone and opens the stage for guidelines regarding the diagnosis of DESD.

## Figures and Tables

**Table 1:** Number of patients with diagnosis of detrusor-external sphincter dyssynergia (DESD) identified by patch electromyogram (EMG), voiding cystourethrogram (VCUG) or both studies by sex of patient.

Test	Male	Female	Total
VCUG	1	9	10
Patch EMG	11	1	12
Both	11	13	24

Total	23	23	46

**Table 2:** Binomial test of patch electromyogram (EMG) and voiding cystourethrogram (VCUG) diagnosis of detrusor-external sphincter dyssynergia (DESD).

Category	*N*	Observed proportion	Test proportion	Exact significance (1-tailed)
Agreement	25	0.54	0.7	0.020
Disagreement	21	0.46		

**Table 3:** Type of neurologic diagnosis in patients diagnosed with detrusor-external sphincter dyssynergia DESD. (Note that some patients may have more than one diagnosis.)

Diagnosis	Number of patients
Multiple sclerosis	13
Spinal cord injury	11
Paraplegia	7
Quadraplegia	4
Spinal disc disease	5
Closed head injury	3
Spinal surgery	3
Spinal stenosis	2
Freidrich's ataxia	1
Spina bifida	1
Focal cortical lesion	1
Myasthenia gravis	1
Other	6
